# High-Alpine Permafrost and Active-Layer Soil Microbiomes Differ in Their Response to Elevated Temperatures

**DOI:** 10.3389/fmicb.2019.00668

**Published:** 2019-04-03

**Authors:** Petra Luláková, Carla Perez-Mon, Hana Šantrůčková, Joel Ruethi, Beat Frey

**Affiliations:** ^1^Forest Soils and Biogeochemistry, Swiss Federal Institute for Forest, Snow and Landscape Research WSL, Birmensdorf, Switzerland; ^2^Faculty of Science, University of South Bohemia, České Budějovice, Czechia

**Keywords:** climate warming, European Alps, permafrost, active soil layer, microcosm, bacterial community, fungal community, microbial functioning

## Abstract

The response of microbial communities to the predicted rising temperatures in alpine regions might be an important part of the ability of these ecosystems to deal with climate change. Soil microbial communities might be significantly affected by elevated temperatures, which influence the functioning of soils within high-alpine ecosystems. To evaluate the potential of the permafrost microbiome to adapt to short-term moderate and extreme warming, we set up an incubation experiment with permafrost and active soil layers from northern and southern slopes of a high-alpine mountain ridge on Muot da Barba Peider in the Swiss Alps. Soils were acclimated to increasing temperatures (4–40°C) for 26 days before being exposed to a heat shock treatment of 40°C for 4 days. Alpha-diversity in all soils increased slightly under gradual warming, from 4 to 25°C, but then dropped considerably at 40°C. Similarly, heat shock induced strong changes in microbial community structures and functioning in the active layer of soils from both northern and southern slope aspects. In contrast, permafrost soils showed only minor changes in their microbial community structures and no changes in their functioning, except regarding specific respiration activity. Shifts in microbial community structures with increasing temperature were significantly more pronounced for bacteria than for fungi, regardless of the soil origin, suggesting higher resistance of high-alpine fungi to short-term warming. Firmicutes, mainly represented by *Tumebacillus* and Alicyclobacillaceae OTUs, increased strongly at 40°C in active layer soils, reaching almost 50% of the total abundance. In contrast, Saccharibacteria decreased significantly with increasing temperature across all soil samples. Overall, our study highlights the divergent responses of fungal and bacterial communities to increased temperature. Fungi were highly resistant to increased temperatures compared to bacteria, and permafrost communities showed surprisingly low response to rising temperature. The unique responses were related to both site aspect and soil origin indicating that distinct differences within high-alpine soils may be driven by substrate limitation and legacy effects of soil temperatures at the field site.

## Introduction

Permafrost is defined as ground material that remains below 0°C for two or more consecutive years and is overlain by seasonally unfrozen soil called the active layer (Pewe, 1995). Permafrost soils represent 25% of the world land area and are mostly located in Arctic, Antarctic, and high-alpine regions (Graham et al., 2012). They constitute a large reservoir of organic carbon, especially in the Arctic (Schuur et al., 2008). Nowadays, thaw of permafrost with global warming has been reported with large transformations of the previously frozen organic carbon and nutrients, which lead to the release of greenhouse gasses (GHG; CO_2_, CH_4_, and N_2_O) (Schuur et al., 2008; Graham et al., 2012; Gobiet et al., 2014; Mackelprang et al., 2016). Despite the hostile conditions in regions where it exists, permafrost harbors a rich microbiota (prokaryotes and fungi) with high functional diversity (Margesin and Miteva, 2011; Jansson and Taş, 2014; Frey et al., 2016; Altshuler et al., 2017) and a large number of unknown taxa (Gilichinsky et al., 2007; Jansson and Taş, 2014; Frey et al., 2016). Permafrost microbes are able to grow and metabolize at subzero temperatures (Gilichinsky et al., 2007; Graham et al., 2012) and can use a wide variety of substrates under either aerobic or anaerobic conditions. Carbon (C) and nitrogen (N) mineralization activities (Yergeau et al., 2007; Samarkin et al., 2010; Zhu et al., 2015), as well as a significant release of GHGs (e.g., 25 to 45 g CO_2_, but also N_2_O and CH_4_), have been reported (Mackelprang et al., 2016; Nikrad et al., 2016), although details of the metabolic processes involved have not been determined.

The soil microbiome in the active layer differs from that in permafrost (Tveit et al., 2014; Frey et al., 2016) and therefore may react differently to increasing temperatures. In permafrost, the release of previously frozen organic matter and increased water availability may significantly promote microbial growth and activity, leading to the subsequent emission of the GHGs CO_2_, N_2_O, and CH_4_ (Knoblauch et al., 2013; Schuur et al., 2015). In addition, microbial activity and the rate of organic matter decomposition have been shown to be site dependent, owing to differences in the carbon quality, moisture and nutrient availability, or redox potential of the soil (Bardgett et al., 2008; Schuur et al., 2008; Gentsch et al., 2018). Moreover, microbial activity is sometimes supported by the reactivation of various ancient microorganisms that have survived, preserved in a dormant state in the frozen soil, for thousands and even millions of years (Gilichinsky et al., 2007). The gradual transformation of permafrost to seasonally frozen soils, followed by deepening of the active layer due to higher temperatures will lead to increasing quantities of soil subjected to freeze-thaw cycles (FTCs) (Gruber et al., 2004; Henry, 2007). FTCs might result in: (i) a shift in the microbial community structure caused by the death of vulnerable microorganisms and (ii) a growth and significant increase in activity of the surviving microorganisms due to the increase in bioavailable C and N from the breakdown of soil aggregates and dead biomass (Schimel and Mikan, 2005; Koponen et al., 2006; Sawicka et al., 2010).

Compared with Arctic permafrost, alpine permafrost is predicted to be more sensitive to climate change (Haeberli et al., 1993) because of its high degree of discontinuity and higher temperatures (>−2°C compared to −10°C in Arctic permafrost; Gruber and Haeberli, 2009). However, the alpine permafrost microbiome is much less understood, and research has been restricted to the Himalayas (Hu et al., 2015), while data from other alpine regions (e.g., the European Alps) are lacking. Further, data that are currently available are mainly related to microbial diversity (Hu et al., 2015), while the functionality of microbial communities and, of utmost importance, their behavior under conditions of climate change (e.g., permafrost thaw and FTCs), remain unknown (Altshuler et al., 2017). Wu et al. (2015) and de Scally et al. (2016) reported that temperature shifts in bacterial composition are closely linked to functional changes in the soil, but there is also evidence for functional redundancy of soil bacteria (Allison and Martiny, 2008). Data on functional changes in fungal communities are scarce (Xiong et al., 2014a). Large uncertainty in predictions of microbial responses to permafrost thawing is also connected with heterogeneity of the environmental conditions, such as differences in topography and in the degree of vegetation coverage (Gruber and Haeberli, 2009). In particular, differences in incoming radiation between southern and northern slope aspects are larger in the European Alps than in the Arctic (Barry, 2008). Strong effects of incoming radiation, namely distinct bacterial and fungal community structures between aspects [north-west (NW) and south-east (SE)] and layers (active versus permafrost), were reported by Frey et al. (2016).

The goals of the present study were to elucidate: (i) the potential of microbial communities to adapt to increasing permafrost temperatures and a seasonally frozen active layer; (ii) their response to a heat shock; and (iii) the effects of slope aspect on the soil microbiome of the active layer and its acclimation responses. We hypothesized that: (i) the microbiome from a seasonally frozen active layer is less responsive, with respect to community structure and functionality, to heat shock and increasing temperatures than the permafrost microbiome; (ii) the soil microbiome from a SE-exposed slope is more adapted to short-term moderate and extreme warming than the microbiome from a NW-exposed slope; and (iii) shifts in community structures are closely linked to functional changes in the soil, with different contributions of bacteria and fungi. To test our hypotheses, we performed a mesocosm experiment under controlled conditions with three different soils from the same location (Frey et al., 2016) but different slope aspects (NW and SE) and different soil depths [active layer from the top soil and 12,000-year-old permafrost (PF) at a soil depth of 160 cm]. Soils were gradually exposed to increasing temperatures (4–40°C) for 26 days, as acclimation, before being exposed to a heat shock of 40°C for 4 days.

## Materials and Methods

### Site and Soil Description

Soils were collected in autumn 2016 on a ridge of the mountain “Muot da Barba Peider” in the eastern Swiss Alps at 2979 m a.s.l., as previously described (Frey et al., 2016). Top-soil samples (5–10 cm soil depth) were taken from the NW and SE flanks of the ridge. The NW slope has continuous permafrost below 1 m depth, and permafrost soil was sampled from a depth of 160 cm. The SE slope is only seasonally frozen. The annual mean soil temperature at 5 cm soil depth was −1.6°C in the NW top soils and +1.7°C in the SE top soils. Minimum and maximum soil temperatures between 2017 and 2018 were −12.0 and +16.6°C for the NW aspect and −3.5 and +29.6°C for the SE aspect. Temperature in permafrost soils at 150 cm soil depth was recorded to be −0.8°C in “Muot da Barba Peider” (Zenklusen Mutter et al., 2010). The regional mean annual precipitation fluctuates around 1500 mm (Meteoschweiz). Vegetation was equally scarce on both sides of the ridge, basically representing barren soil with some rare individual occurrences of plants, in particular *Poa*, *Cerastium*, and *Jacobea* spp. (Frey et al., 2016). In order to evaluate the influence of different soil parameters and of nutrient status on the response of the microbial community to warming, an incubation experiment was set up with soils from the three different origins. Fresh top-soil samples were kept at 4°C and permafrost samples were kept at −1.5°C until the incubation experiment started. Two days before the experiment started, soils were sieved through a 2 mm mesh (after thawing at 4°C for the PF sample) and homogenized.

### Experimental Design

A 39 day long incubation experiment was set up similarly to in previous laboratory-based warming studies (Bárcenas-Moreno et al., 2009; Wu et al., 2015). The experiment was composed of two stages: (i) *acclimation* of the soils at different temperatures from 4°C (control) up to 40°C followed by (ii) *heat shock* response assessment by exposing the soil to 40°C. A 60 g aliquot of wet soil of each origin (NW, SE, and PF) was weighed into 250 ml glass jars, with a cotton wool permeable lid to allow for aeration, and incubated in a growth chamber set to different temperature regimes. Four different temperatures (4°C control, 15, 25, and 40°C) were used for active layer samples and two (4 and 25°C) for the PF samples. In the acclimation stage, the soils were left at 4°C or exposed to gradually increasing temperatures for 14 days reaching 15, 25, and 40°C (increase of +1, 2, and 3°C per day, respectively), followed by 12 days of stabilization. After stabilization, one part of each soil sample (8 g) was used for destructive analyses and the other part (52 g) was exposed to 40°C for 4 days, followed by a gradual temperature decrease back to 4°C over 9 days (decrease −6°C day^−1^ for all soils) and then analyzed. There were four replicates for each soil origin and treatment, resulting in a total of 40 glass jars; one jar containing a 4°C PF sample was lost during the experiment. Soil moisture in all samples was monitored gravimetrically during the whole experiment and the samples were watered with autoclaved tap water every second day. The 4°C acclimation sample was used as a control for the heat shock part of the experiment, as a previous trial incubation showed only minor changes in the nutrient status and microbial composition between 3 and 5 weeks of incubation at 4°C (data not presented). At the end of both the acclimation period and the heat shock period, soil was analyzed for DNA content and microbial composition, respiration, potential enzyme activity, and carbon utilization patterns.

### Soil Chemical and Microbial Parameters

Dissolved organic carbon (DOC) and dissolved nitrogen (DN) were analyzed in a MilliQ water extract (H_2_O:soil = 5:1 v/w, 18 h, vertical shaker at room temperature), passed through paper filters (Nr. 0790 1/2, Schleicher & Schuell, Dassel, Germany) and analyzed on a Shimadzu TOC-500 apparatus (Shimadzu, Kyoto, Japan).

Soil basal respiration was determined using an EGM4 gas analyzer (PP System). Each glass jar was aerated and sealed with butyl rubber stoppers. Sealed flasks were measured immediately after sealing and then returned to a given incubation temperature and measured again after 20 h. The final respiration rate was converted from the headspace CO_2_ change in ppm to μg C g^−1^ h^−1^ and then normalized, using the DNA amount as a proxy for microbial biomass, to μg C g DNA^−1^ h^−1^ (Anderson and Martens, 2013) to calculate the specific respiration activity (SRA) (Hartman and Richardson, 2013).

Potential activities of extracellular enzymes were measured for five soil enzymes responsible for organic carbon, nitrogen, and phosphorus acquisition. A standard fluorometric technique was used (Marx et al., 2001) with a modification according to Bárta et al. (2014). The activity of extracellular enzymes was determined in unbuffered water extracts. Briefly, 0.5 g of soil was homogenized in 50 ml of MilliQ water using 4 min ultrasonication (HF-Frequency 35 kHz, Bandelin, Berlin, Germany). The soil suspensions (200 μl) were then transferred to a 96-well microplate. Subsequently, 50 μl of substrate labeled with 4-methylumbelliferone (MUB) or 7-amino-4-methylcoumarin (AMC) was added. Standard curves were measured with MUB/AMC in soil slurries for every sample separately. Concentrations of MUB standard curves were 0.5, 2.5, and 5 μM and concentrations of AMC standard curves were 0.1, 0.5, and 1 μM. Microplates were incubated at room temperature for 2 h. All fluorescence measurements were carried out with the microplate reader INFINITE F200 (Tecan, Männedorf, Switzerland) using an excitation wavelength of 360 nm and an emission wavelength of 465 nm. Enzyme activities were expressed as nmol h^−1^ g^−1^dw. Results are presented as the sum of all potential activities of five measured extracellular enzymes and as the ratio of carbon-acquiring enzymes (C enzymes, the sum of β-glucosidases and β-xylosidases), nitrogen-acquiring enzymes (N enzymes, the sum of leucine aminopeptidases and chitinase) and phosphorus-acquiring enzymes (P enzymes, the sum of phosphatases) (Čapek et al., 2015).

Carbon utilization patterns of the soil microbial community were determined using Biolog EcoPlates^TM^ (Biolog Hayward, CA, United States), which contain 31 carbon sources and a control (water) in triplicate in a 96-well plate. Soil suspensions were prepared according to Zimmermann and Frey (2002). Briefly, 5 g triplicates of fresh soil samples were shaken in 50 ml 0.9% NaCl solution for 2 h in a vertical shaker and left for 10 min to settle in order to clear the supernatant, which was then diluted 10-fold to obtain a final dilution of 10^−2^. A 125 μl aliquot was dispensed into each well of the Biolog plates. The microplates were incubated in darkness at 25°C for 72 h. The optical density (OD) of each well was read at 590 nm with the INFINITE F200 (TECAN). After subtracting the OD of a control well, the mean across 31 different substrates was calculated as the average well color development (AWCD). AWCD thus represents the C use capacities of the 31 substrates and was used to calculate functional diversity (Shannon index) and to perform an ordination based on Bray-Curtis dissimilarity distances (Wu et al., 2015).

### DNA Extraction, PCR Amplification, and Illumina MiSeq Sequencing

Total genomic DNA was extracted from approximately 0.5 g soil per sample using the Power Soil DNA Isolation kit (Qiagen, Hilden, Germany) according to the manufacturer’s instructions. Because of the small amount of DNA recovered from permafrost soils (PF), four DNA extractions were made from the same sample, pooled and collected on a single column in order to obtain a concentrated DNA eluate. DNA was quantified with PicoGreen (Invitrogen, Carlsbad, CA, United States). The V3–V4 region of the bacterial small-subunit (16S) rRNA gene and the internal transcribed spacer region 2 (ITS2) of the eukaryotic (fungal groups and some groups of protists and green algae) ribosomal operon were PCR amplified from 5 ng of DNA template, using primers and conditions previously described (Frey et al., 2016). PCRs were run in triplicate, pooled and purified using Agencourt Ampure XP (Beckman Coulter, Beverly, MA, United States). Bacterial and fungal amplicon pools were sent to the Génome Québec Innovation Centre at McGill University (Montreal, QC, Canada) for barcoding using the Fluidigm Access Array technology (Fluidigm) and paired-end sequencing on the Illumina MiSeq v3 platform (Illumina Inc., San Diego, CA, United States). Raw sequences have been deposited in the NCBI Sequence Read Archive under the BioProject accession number PRJNA497433.

### Sequence Quality Control, OTU Clustering, and Taxonomic Assignments

Quality filtering, clustering into operational taxonomic units and taxonomic assignment were performed as previously described (Frey et al., 2016). A customized pipeline largely based on UPARSE (Edgar, 2013; Edgar and Flyvbjerg, 2015) and implemented in USEARCH v. 9.2 (Edgar, 2010) was used. Briefly, paired-end reads were merged using the USEARCH fastq mergepairs algorithm (Edgar and Flyvbjerg, 2015). Substitution errors arising because of phasing events during Illumina sequencing were corrected by applying Bayes Hammer (Nikolenko et al., 2013). PCR primers were removed using Cutadapt (Martin, 2011) and reads were quality filtered using the USEARCH fastq filter function. Subsequently, sequences were dereplicated, singletons were discarded, and sequences were clustered into OTUs based on 97% identity (Edgar, 2013). OTU centroid sequences were checked for the presence of ribosomal signatures with Metaxa2 (Bengtsson-Palme et al., 2015) or ITSx (Bengtsson-Palme et al., 2013). For taxonomic classification of the OTUs, corresponding centroid sequences were queried against selected reference databases using the naïve Bayesian classifier (Wang et al., 2007) implemented in MOTHUR (Schloss et al., 2009) and a minimum bootstrap support of 60%. Prokaryotic 16SV3V4 sequences were queried against the GREENGENES database (DeSantis et al., 2006), whereas eukaryotic ITS2 sequences were first queried against a custom-made ITS2 reference database retrieved from NCBI GenBank and sequences assigned to fungi were subsequently queried against the fungal ITS database UNITE (Abarenkov et al., 2010). Prokaryotic centroid sequences identified as originating from organelles (chloroplast, mitochondria), as well as eukaryotic centroid sequences identified as originating from soil animals (metazoa) or plants (viridiplantae, except green algae), or of unknown eukaryotic origin, were removed from downstream analyses.

### Statistical Analyses

Statistical analyses were performed with the statistical package R version 3.4.0 (R Core Team, 2017), except for the unconstrained principal component analysis (PCA) of basic soil properties and non-metric multidimensional scaling analyses (NMDS) of Biolog EcoPlates, which were performed in CANOCO for Windows 5.0 (Ter Braak and Šmilauer, 2012). Basic soil properties, microbial functional characteristics, alpha-diversity and bacterial and fungal phyla changes were analyzed using generalized linear models with assumed Gamma distribution (link = log) implemented in the R package *stats*. The listed parameters were assessed separately after the acclimation and after the heat shock. Pairwise significant differences between the temperature groups were consequently evaluated using Tukey’s HSD *post hoc* tests coded in the R package *multcomp*. Microbial alpha-diversity is presented as Richness, Evenness, and Shannon Index. The indices were calculated using the R package *phyloseq* from evenly rarefied OTU matrices (29,775 sequences for bacteria and 34,939 sequences for fungi) and boxplots of the Shannon index were plotted in R. Rarefied OTUs were also used to calculate changes in bacterial and fungal phyla, which were plotted in R. Estimates of beta-diversity were measured on Bray-Curtis dissimilarity distances of square-root transformed relative abundances of OTUs per sample and displayed with canonical analysis of principal coordinates (CAP). The results were then evaluated with permutational multivariate analysis of variance (PERMANOVA) and permutational analysis of multivariate dispersion with 9,999 permutations with the functions “adonis” and “betadisper” from the R package *vegan* (v2.5.3; Oksanen et al., 2018). For the acclimation experiment the OTUs that changed the most from one temperature level to another were determined for each soil origin. All OTUs were filtered for relative abundance >0.001, and then their log2-fold changes (Rime et al., 2016) compared to the 4°C control were calculated for each temperature (15, 25, and 40°C). For each temperature, the five OTUs that increased or decreased the most were visualized with a *ggplot2* heat map (15 OTUs were selected for PF soils tested at only two temperatures). OTUs were sorted according to phylum, with Proteobacteria and Ascomycota further classified to class level. Results were then complemented by bar plots showing the sum of the relative abundance of the OTUs for all acclimation temperatures to verify the importance of particular taxa in the changes. To evaluate the coupling between functional changes and microbial community structure, simple linear regressions were built by fitting the Bray-Curtis dissimilarity distances for the bacterial and fungal communities (calculated with the R package *ecodist*) against the distances of all 31 carbon substrates and potential enzyme activities, both together and separately, similarly to Wu et al. (2015). Regression *R*^2^ values were then bootstrapped using the R package *boot* with a 5% confidence interval, and plotted separately for fungi and bacteria to investigate which functional changes were driven by bacteria and which by fungi.

## Results

### Soil Parameters

**FIGURE 1:**
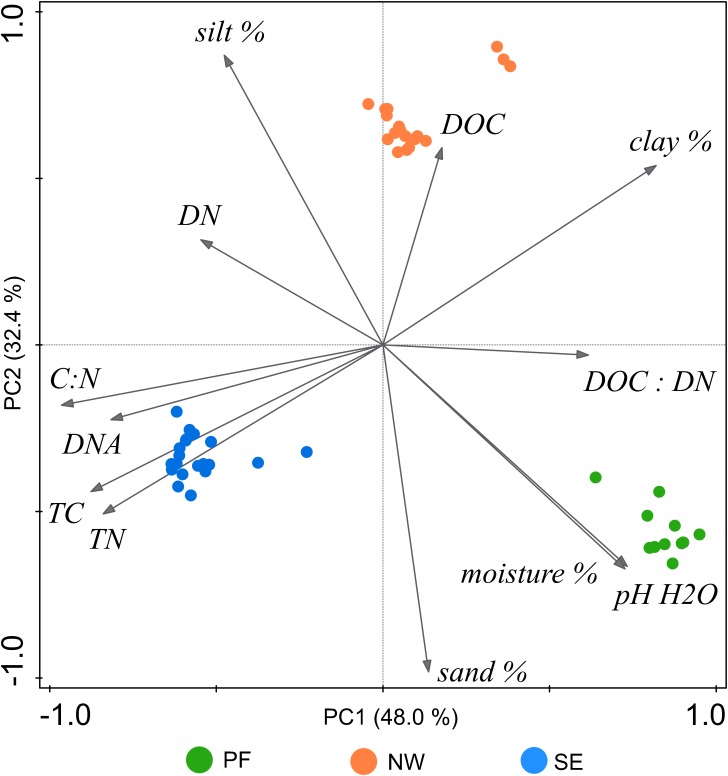
Unconstrained PCA ordination of characteristics (at the time of incubation) of soils from the three investigated origins on “Muot-da-Barba-Peider” in the eastern Swiss Alps. DNA, DNA content; DOC, dissolved organic carbon; DN, dissolved nitrogen; TC, total soil organic carbon; TN, total soil nitrogen; pH H_2_O, pH in water extract; moisture %, gravimetric soil moisture; sand, silt, and clay %, percentage of each soil texture components.

Soil characteristics clearly differed among the three soil origins (PF, permafrost soil; NW, active layer soil on north-west exposed slope; SE, active layer soil on south-east exposed slope, Table 1 and Figure 1). Compared with NW soils, PF soils had a higher pH and lower organic carbon content, with a lower C/N ratio and lower DOC. SE soils had the lowest pH (4.61) and the highest organic carbon but lower DOC than NW soils (Table 1).

**Table 1:** Basic properties of the investigated soils.

	pH (H_2_O)	Moisture %	C:N	TC %	DOC (μgC g^−1^dw)	DN (μgN g^−1^dw)	Sand %	Silt %	Clay %
	***F*_2,8_ = 1697^∗∗∗^**	***F*_2,8_ = 291^∗∗∗^**	***F*_2,8_ = 210^∗∗∗^**	***F*_2,8_ = 931^∗∗∗^**	***F*_2,8_ = 16.8^∗∗^**	***F*_2,8_ = 0.37^NS^**	***F*_2,8_ = 939^∗∗∗^**	***F*_2,8_ = 172^∗∗∗^**	***F*_2,8_ = 52.2^∗∗∗^**
**PF**	7.44 (0.01) a	20.4 (0.75) a	2.83 (0.17) c	0.06 (0.00) c	53.1 (8.86) b	1.36 (0.54)	85.2 (0.10) a	11.2 (0.20) a	3.53 (0.09) a
**NW**	6.50 (0.07) b	9.07 (0.00) c	6.83 (0.41) b	0.14 (0.01) b	137 (3.97) a	1.11 (0.05)	80.5 (0.04) c	15.9 (0.15) c	3.60 (0.12) a
**SE**	4.61 (0.03) c	10.7 (0.08) b	13.6 (1.14) a	0.90 (0.02) a	47.9 (4.41) b	1.89 (0.83)	83.8 (0.15) b	14.0 (0.29) b	2.17 (0.21) b

*Statistics: F-ratio test (F) determined by a GLM model with a Gamma distribution to assess differences between the soils. Asterisks indicate significant differences with ^∗∗∗^p < 0.001, ^∗∗^p < 0.01. Soil properties: TC, total organic carbon; TN, total nitrogen; DOC, dissolved organic carbon; DN, dissolved nitrogen. Soil origins: PF, permafrost; NW, active layer on north-western slope; SE, active layer on south-eastern slope. Values in brackets indicate mean ± standard error (within each soil, *n* = 3). Different lowercase letters indicate significant differences (*p* < 0.05) between soils (multiple comparisons using Tukey *post hoc* test).*

### Acclimation Experiment

#### Functional Responses to Warming

**FIGURE 2:**
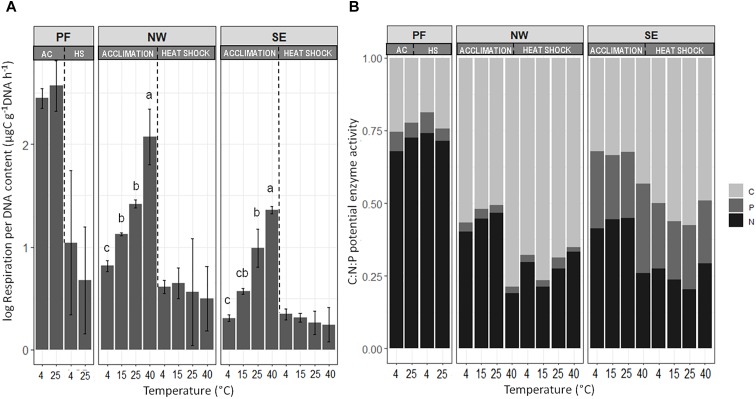
Changes in **(A)** specific respiration activity and **(B)** the C:N:P ratio of acquiring enzymes at the end of the incubation. Letters indicate significant differences (*p* < 0.05) between treatments (temperatures) for acclimation (AC) and heat shock (HS), assessed separately with Tukey *post hoc* tests. Enzymes are plotted in relative proportions of carbon – (C), nitrogen – (N), and phosphorus – (P) acquiring enzymes. Soil origins: PF, permafrost; NW, active layer on north-western slope; SE, active layer on south-eastern slope. Temperatures: PF soils 4 and 25°C; NW and SE soils 4, 15, 25, and 40°C.

Microbial functioning was significantly affected by increases in temperature, and the temperature response of PF soils differed from that in NW and SE soils. Temperature responses of microbial processes in the active soil layers were partly slope dependent (Table 2). At 4°C (control), DNA content was lower by two orders of magnitude in PF than in the active layer soils, and the sum of enzyme activities was lower by more than one order of magnitude (Supplementary Table S1). In contrast, SRA was two orders of magnitude higher at 4°C in PF soils than in NW and SE soils (Figure 2 and Supplementary Table S1). SE soils had lower SRA and more DNA, but similar enzyme activities than NW soils (Figure 2 and Supplementary Table S1). Pair-wise tests showed no significant response of DNA content, SRA and enzyme activities to temperature between 4 and 25°C in PF soils and an increase in SRA in SE and NW soils, that responded strongly to temperature change when temperature rose to 40°C (Figure 2 and Supplementary Table S1). At 40°C, DNA content dropped strongly and SRA increased in both NW and SE soils, but the response was more pronounced in NW (Supplementary Table S1 and Figure 2). In line with this observation, enzyme activities only decreased significantly with increasing temperatures in SE soils (Supplementary Table S1). The main effect of temperature increase at 40°C was in the proportion of activity of C-acquiring enzymes out of the total enzyme activity, which increased in both NW and SE soils at the expense of N-acquiring enzymes (Figure 2). The proportion of P-acquiring enzymes likewise increased with increasing temperature in SE soils (Supplementary Table S1 and Figure 2). The functional diversity (Shannon index) of C utilization patterns did not reveal any distinct changes with increasing temperature (data not shown), whereas the NMDS patterns were significantly altered with higher temperatures for both active layer soils but not for the PF soils (Supplementary Figure S1). Utilization of C substrates shifted from recalcitrant to more easily degradable forms, and the shift was more pronounced in NW soils (pseudo-F_PERMANOVA_ = 3.65, *p* < 0.001) than in SE soils (pseudo-F_PERMANOVA_ = 7.71, *p* < 0.001; Supplementary Figure S1).

**Table 2:** Main effects of soil origin, temperature, and their interactions on DNA content, specific respiration activity, potential enzymatic activities and bacterial and fungal Shannon-diversity Index.

	DNA	SRA	SUM enz	Cenz	Nenz	Penz	Alpha-diversity (bacteria)	Alpha-diversity (fungi)

	ng g^−1^dw	μg C g^−1^ DNA h^−1^	nmol h^−1^ g^−1^dw		% of SUM enz		Shannon	Shannon
**Main effects (acclimation)**							
Soil origin	*F*_2,38_ = 135^∗∗∗^	*F*_2,38_ = 24.4^∗∗∗^	*F*_2,38_ = 178^∗∗∗^	*F*_2,38_ = 55.0^∗∗∗^	*F*_2,38_ = 20.9^∗∗∗^	*F*_2,38_ = 299^∗∗∗^	*F*_2,38_ = 13.6^∗∗∗^	*F*_2,38_ = 15.9^∗∗∗^
Temperature	*F*_3,38_ = 3.70^∗^	*F*_3,38_ = 4.25^∗^	*F*_3,38_ = 1.37^NS^	*F*_3,38_ = 4.61^∗∗^	*F*_3,38_ = 21.2^∗∗^	*F*_3,38_ = 0.48^NS^	*F*_3,38_ = 7.67^∗∗∗^	*F*_3,38_ = 1.52^NS^
Soil × temperature	*F*_9,38_ = 69.3^∗∗∗^	*F*_9,38_ = 65.2^∗∗∗^	*F*_9,38_ = 56.3^∗∗∗^	*F*_9,38_ = 25.9^∗∗∗^	*F*_9,38_ = 36.1^∗∗∗^	*F*_9,38_ = 103^∗∗∗^	*F*_9,38_ = 83.2^∗∗∗^	*F*_9,38_ = 22.8^∗∗∗^
**Main effects (heat shock)**							
Soil origin	*F*_2,38_ = 96.5^∗∗∗^	*F*_2,38_ = 9.13^∗∗∗^	*F*_2,38_ = 172^∗∗∗^	*F*_2,38_ = 62.1^∗∗∗^	*F*_2,29_ = 75.8^∗∗∗^	*F*_2,29_ = 187^∗∗∗^	*F*_2,38_ = 6.68^∗∗^	*F*_2,38_ = 0.87^NS^
Temperature	*F*_3,38_ = 0.76^NS^	*F*_3,38_ = 0.71^NS^	*F*_3,38_ = 1.39^NS^	*F*_3,38_ = 1.45^NS^	*F*_3,38_ = 0.08^NS^	*F*_3,38_ = 0.04^NS^	*F*_3,38_ = 12.3^∗∗∗^	*F*_3,38_ = 4.89^∗∗^
Soil × temperature	*F*_9,38_ = 47.0^∗∗∗^	*F*_9,38_ = 2.21^NS^	*F*_9,38_ = 65.5^∗∗∗^	*F*_9,38_ = 13.6^∗∗∗^	*F*_9,38_ = 19.3^∗∗∗^	*F*_9,38_ = 75.0^∗∗∗^	*F*_9,38_ = 31.1^∗∗∗^	*F*_9,38_ = 3.00^∗^

**FIGURE 3:**
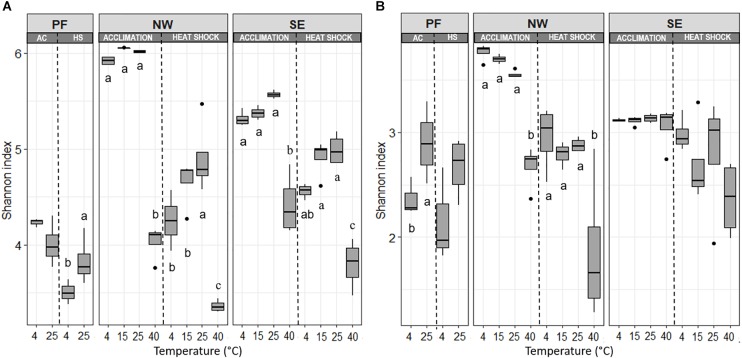
Changes in **(A)** bacterial and **(B)** fungal Shannon index with increasing temperatures, presented separately for acclimation and for heat shock. Horizontal lines represent the median and boxes represent the range between first and third quartiles. Vertical lines represent the maximal and minimal values and points represent extremes. Different letters indicate significant differences (*p* < 0.05) between temperature treatments for acclimation and heat shock assessed separately with Tukey *post hoc* tests. Soil origins: PF, permafrost; NW, active layer on north-western slope; SE, active layer on south-eastern slope. Temperatures: PF soils 4 and 25°C; NW and SE soils 4, 15, 25, and 40°C.

*Statistics: F-ratio test (F) determined by a GLM model with a Gamma distribution to assess the main effects of soil origin, temperature and soil origin × temperature interactions separately during acclimation and after a heat shock. Asterisks indicate significant differences between treatments with ^∗∗∗^p < 0.001, ^∗∗^*p* < 0.01, ^∗^p < 0.05, and NS not significant. Means and *post hoc* test significances are reported in Figure 2 and Supplementary Table S1 for microbial activities and in Figure 3 and Supplementary Table S2 for alpha-diversity. Soil origin (PF, NW, SE), temperature (PF soils 4 and 25°C, NW and SE soils 4, 15, 25, and 40°C). Potential activities of enzymes: Cenz, carbon processing enzymes β-glucosidases and β-xylosidases; Nenz, aminopeptidases and chitinases; Penz, phosphatases; SUM enz, sum of Cenz; Nenz and Penz; SRA, specific respiration activity, calculated as basal respiration normalized to the DNA content; dw, soil dry weight.*

#### Changes in Bacterial and Fungal Community Structure in Response to Warming

After quality filtering, 29,775 (±3,773) prokaryotic 16S rRNA sequences per sample were obtained (2,649,945 sequences total), of which 5,722 OTUs were identified, and 34,939 (±8,800) fungal ITS2 sequences per sample were obtained (3,227,931 total), of which 1812 OTUs were identified. Based on a sequence similarity threshold of 97%, the number of OTUs for each sample ranged from 634 to 1,941, with 99.6% of the OTUs representing bacteria and 0.4% representing archaea. The number of fungal OTUs for each sample ranged from 115 to 271. The percentage of bacterial OTUs occurring under all the acclimation temperatures (36%) was higher than the percentage present only under one temperature treatment (21%), and the percentage of unique OTUs was similar for all temperature treatments (20–28%). Likewise, a higher percentage of fungal OTUs was found under all temperature treatments (46%) than only at one temperature (19 %), but the largest percentage of fungal OTUs unique to a single temperature was found for the 40°C treatment (40%). The most abundant phyla in the 4°C controls differed between the soil origins (Supplementary Table S3). In PF soils Actinobacteria, Proteobacteria and Verrucomicrobia were the most abundant phyla, with relative abundances of 38.6, 18.4, and 14.3%, respectively; in NW soil Proteobacteria, Verrucomicrobia, and Bacteroidetes were most abundant (26.0, 14.6, and 14.2%, respectively); and in SE soils Proteobacteria, Saccharibacteria and Chloroflexi were most abundant (25.2, 17.6, and 17.2%, respectively). Fungi were represented predominantly by Basidiomycota in PF soils, with a relative abundance of 68.3%, and by Ascomycota in NW and SE soils (77.1 and 48.5%, respectively, Supplementary Table S4).

Soil origin and temperature treatment strongly affected bacterial alpha-diversity (Figure 3, Table 2, and Supplementary Table S2). At 4°C, PF soils had a smaller number of observed species (S_obs_) and lower Shannon index values than soils from the active layers. In general, alpha-diversity was higher in NW than in SE soils. Shannon index and evenness did not change significantly between 4 and 25°C in any soil, but they both dropped strongly at 40°C, with a more pronounced decrease in NW than in SE soils (Figure 3 and Supplementary Table S2). Fungal alpha-diversity was less affected by the increase in temperatures than bacterial alpha-diversity (Figure 3 and Supplementary Table S2). Similar to bacterial alpha-diversity, the lowest S_obs_ and Shannon index for fungi at 4°C was found in PF soils and the highest in NW soils (Figure 3 and Supplementary Table S2). Fungal Shannon index and evenness increased from 4 to 25°C in PF soils. No changes in fungal Shannon index and evenness with increasing temperatures were observed for SE soils, even at 40°C, but a decrease in both parameters was observed for NW soils with a drastic drop at the highest temperature (Figure 3 and Supplementary Table S2).

**Table 3:** Main effects of soil origin, temperature and their interactions on bacterial and fungal beta-diversity.

	Bacteria			Fungi		
	PERMANOVA (pseudo-F)	Explained variability	Dispersion	PERMANOVA (pseudo-F)	Explained variability	Dispersion
**Main effects (acclimation)**						
Soil origin	90.2^∗∗∗^	68.7%	1.24^NS^	76.0^∗∗∗^	74.6%	1.85^NS^
Temperature	11.1^∗∗∗^	12.7%	2.09^NS^	4.62^∗∗∗^	6.80%	4.95^∗∗^
Soil × temperature	4.98^∗∗∗^	7.59%	27.9^∗∗∗^	2.24^∗^	4.40%	7.14^∗∗∗^
**Main effects (heat shock)**						
Soil origin	61.3^∗∗∗^	65.4%	3.20^∗^	47.2^∗∗∗^	67.6%	5.72^∗∗^
Temperature	7.24^∗∗∗^	11.6%	1.54^NS^	3.21^∗∗∗^	6.87%	4.74^∗∗^
Soil × temperature	3.51^∗∗∗^	7.50%	7.86^∗∗∗^	1.71^NS^	4.87%	5.97^∗∗∗^
**PF**						
Acclimation	1.69^∗^	25.3%	6.73^NS^	1.17^NS^	19.0%	3.48^NS^
Heat shock	3.65^∗∗∗^	51.1%	3.97^NS^	1.49^∗^	29.8%	3.00^NS^
**NW**						
Acclimation	10.2^∗∗∗^	71.7%	102^∗∗∗^	4.42^∗∗∗^	52.5%	2.95^NS^
Heat shock	9.74^∗∗∗^	72.2%	20.8^∗∗∗^	4.93^∗∗∗^	56.8%	13.6^∗∗∗^
**SE**						
Acclimation	8.60^∗∗∗^	68.3%	3.34^∗^	4.20^∗∗∗^	51.2%	3.33^∗^
Heat shock	8.62^∗∗∗^	69.7%	12.5^∗∗∗^	4.55^∗∗^	54.8%	1.35^NS^

*Statistics: pseudo-F-test (pseudo-F) and explained variability determined by PERMANOVA (number of permutations = 9,999) to assess the main effects of soil origin, temperature and soil origin × temperature and their interactions separately during acclimation and after a heat shock. Asterisks indicate significant differences between treatments with ^∗∗∗^p < 0.001, ^∗∗^p < 0.01, ^∗^p < 0.05, and NS not significant. Soil origin (PF, NW, and SE), temperature (PF soils 4 and 25°C, NW and SE soils 4, 15, 25, and 40°C). Significant differences were also tested by an analysis of homogeneity of group dispersion (Dispersion). Main effects are given, followed by effects within each soil origin.*

**FIGURE 4:**
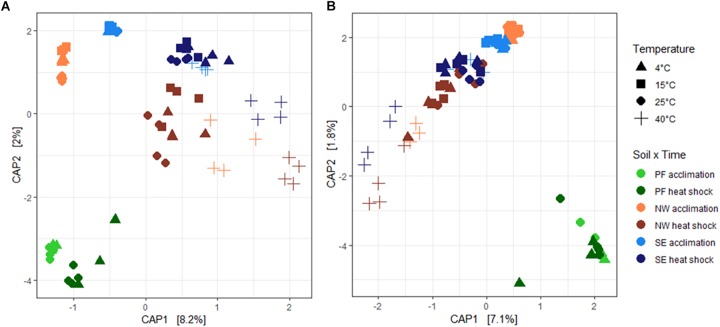
Changes in **(A)** bacterial and **(B)** fungal community structures shown by canonical analysis of principal coordinates (CAP) constrained by temperature. Each ordination shows changes for all soil origins (PF, NW, and SE) and temperatures. Soil origins: PF, permafrost; NW, active layer on north-western slope; SE, active layer on south-eastern slope. Temperatures: PF soils 4 and 25°C; NW and SE soils 4, 15, 25, and 40°C. The acclimation duration was 26 days.

Soil origin explained the highest percentage of variability in bacterial and fungal beta-diversity (68.7 and 74.6%, respectively, Table 3). Similar to alpha-diversity, beta-diversity was more sensitive to increased temperatures for bacteria than for fungi. Temperature explained twice as much variability in bacterial community structures (12.7%) than in fungi, which also varied more in their response (6.8% with significant dispersion). Overall, CAP ordination plots showed that the microbial community structures at temperatures up to 25°C clustered together but shifted strongly at 40°C and after heat shock stress (Figure 4). This shift was less pronounced for PF soils than for the NW and SE active layer soils, in which acclimation and heat shock responses were comparable.

#### The Taxa Most Responsive to Increases in Temperature

**FIGURE 5:**
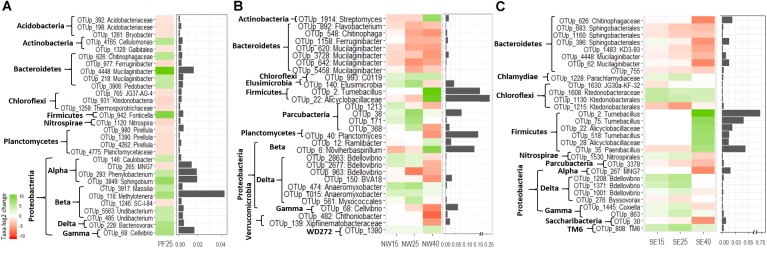
The most pronounced changes in the relative abundance (log2-fold change) of bacterial OTUs during the incubation for **(A)** PF soil: 4°C compared with 25°C; **(B)** NW soil: 4°C compared with 15, 25, and 40°C; and **(C)** SE soil: 4°C compared with 15, 25, and 40°C. OTU names are given at the deepest successful classification and then grouped according to phylum. Proteobacteria are defined to the class level. Changes are displayed by a red (negative) to green (positive) color range and bar plots show relative abundance (%) during acclimation.

Despite a highly inconsistent response of phyla to warming between and within soil origins, the acclimation experiment revealed some clear patterns, which are shown at the phylum level (for bacteria and fungi separately) in Supplementary Tables S3, S4 and at the lowest classified level in Figure 5, 6. Saccharibacteria was the only bacterial phylum that decreased in relative abundance with warming across all samples. Phylum changes in PF soils were mostly not significant, and analyses of the most responsive taxa only showed an increase in some OTUs within Alphaproteobacteria (e.g., genus *Sphingobium*) and Betaproteobacteria (*Massilia*, *Undibacterium*, and in particular *Methylotenera*), as well as an increase in Bacteroidetes, represented mainly by *Mucilaginibacter* (Figure 5). Contrary to this, in both SE and NW active layer soils, there was a pronounced decrease in OTUs within Bacteroidetes (also mainly represented by *Mucilaginibacter* and Sphingobacteriales). In both active layer soils, Planctomycetes, and in particular Deltaproteobacteria, represented mainly by *Bdellovibrio*, either did not change or increased until 25°C and then decreased at 40°C (Figure 5 and Supplementary Table S3). We observed a pronounced increase in sporulating Firmicutes, represented mainly by *Tumebacillus*, and in OTUs within Alicyclobacillaceae at 40°C in NW and SE soils. In particular, Firmicutes strikingly reached up to 50% of the total abundance during acclimation to elevated temperatures in the NW soils (Supplementary Table S3). In both active layer soils Chloroflexi, represented by Ktedonobacterales, also increased with warming, although only slightly (Figure 5 and Supplementary Table S3).

**FIGURE 6:**
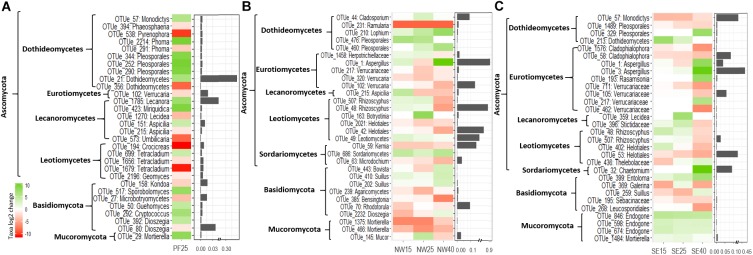
The most pronounced changes in the relative abundance (log2-fold change) of fungal OTUs during the incubation for **(A)** PF soil: 4°C compared with 25°C; **(B)** NW soil: 4°C compared with 15, 25, and 40°C; and **(C)** SE soil: 4°C compared with 15, 25, and 40°C. OTU names are given at the deepest successful classification and then grouped according to phylum. Ascomycota are defined to the class level. Changes are displayed by a red (negative) to green (positive) color range and bar plots show relative abundance (%) during acclimation.

Fungal phyla did not experience any significant changes with increasing temperatures in PF soils and did not show any consistent patterns in SE and NW soils (Supplementary Table S4). Most of the phyla showed highly variable responses between and within soil origins. The most pronounced increase with warming across soils was observed for *Aspergillus*, which was very abundant in SE and NW soils at 40°C, and in Dothideomycetes with many OTUs assigned to Pleosporales (Figure 6). Contrary to this, in both active layer soils, increased temperatures caused a decrease in some OTUs within Verrucariaceae and Helotiales. Notably, *Rhizoscyphus* OTUs increased until 25°C and then dropped strongly (Figure 6). Basidiomycota and Mucoromycota did not exhibit clear patterns of change between 4 and 25°C (Supplementary Table S4). However, the latter decreased significantly in both SE and NW active layer soils at 40°C. In particular, *Mortierella* OTUs, within Mucoromycota, decreased in relative abundance in the active layer soils with the increase in temperatures while it increased in the PF soils (Figure 6).

#### Functional and Phylogenetic Similarities

Shifts in bacterial and fungal community structures during acclimation based on Bray-Curtis dissimilarity distances were plotted against changes in potential enzyme activities and carbon utilization patterns for each soil origin separately, resulting in 12 linear regressions (Supplementary Figure S2). All linear regressions showed significant relationships between variables in both active layer soils but not in PF soil (Supplementary Figure S2). Regressions revealed distinct variation in functional relationships according to soil origin, but the relationship of functional and phylogenetic similarities was not significantly different for bacteria and fungi. The variability of community changes explained by functional parameters was clearly larger in the soils experiencing higher temperatures in field conditions (SE > NW > PF, Supplementary Figure S2).

### Heat Shock Experiment

After acclimation to increasing temperatures, all soil samples were exposed to a 40°C heat shock for 4 days, then subjected to a gradual decrease in temperature back to 4°C over 9 days. Heat shock did not lead to any significant changes in DNA content or potential enzyme activities in PF soils but caused a significant decrease in SRA (Supplementary Table S1). In NW and SE active layer soils, DNA content decreased strongly after heat shock, whereas the effect was less pronounced in SE than in NW soils acclimated to 4, 15, and 25°C. After heat shock, SRA in the NW and SE soils returned to the levels of control (4°C) soils (Figure 2). The sum of potential enzyme activities remained unchanged in the NW soils but dropped significantly in the SE soils (Supplementary Table S1). Investment in C-acquiring enzymes increased at the expense of N-acquiring enzymes in both NW and SE soils (Figure 2).

Bacterial S_obs_, Shannon index and evenness decreased with the heat shock treatment compared with the values in control soils, and this reduction was more pronounced in NW and SE soils than in PF soils (Figure 3 and Supplementary Table S2). For the active layer soils, samples acclimated to 40°C (before heat shock) showed a more pronounced decline in alpha-diversity than soils acclimated to 4–25°C (Figure 3 and Supplementary Table S2). Changes in fungal alpha-diversity were overall not significant except for NW soils, in which a strong drop in S_obs_, Shannon index and evenness occurred after heat shock, especially in the 40°C acclimated soils (Figure 3 and Supplementary Table S2). In the SE soils, only the decrease in S_obs_ was significant. After the heat shock, beta-diversity of the active layer soils acclimated to 4, 15, and 25°C clustered with the soils acclimated to 40°C (before heat shock). However, soils acclimated to 40°C showed another shift (Figure 4). These shifts were again more pronounced for bacteria than for fungi, and temperature explained a similar amount of the variability as for the acclimation experiment, except for PF (Table 3). In PF soils subjected to heat shock, temperature explained 51 and 30% of the variability in bacterial and fungal beta-diversity, respectively, which was about twice the variability explained in the acclimation experiment (25% for bacteria and 19% for fungi). This suggest a stronger influence of the heat shock on the PF soils than on the NW and SE soils.

Heat shock induced a general decrease in the relative abundance of Saccharibacteria and Elusimicrobia in all soil samples (Supplementary Table S3). In contrast, the relative abundance of Firmicutes increased distinctly in all soils and Chloroflexi also became a considerable part of the bacterial community, especially in the SE soil. Bacteroidetes increased in the PF soils after the heat shock reaching 8% of the total abundance. The relative abundance of Alphaproteobacteria acclimated at 40°C significantly decreased after the heat shock in the NW and SE soils, whereas the relative abundance of Betaproteobacteria strongly increased by the heat shock treatment after the acclimation temperature of 40°C reaching 19.4 and 18.3% in the NW and SE soil, respectively (Supplementary Table S3). In contrast, fungal communities in the PF soil were relatively unaffected by the heat shock, with no phyla showing a significant change in relative abundance (Supplementary Table S4). On the other hand, the predominance of Ascomycota in the NW and SE soils increased at the expense of Basidiomycota, Mucoromycota, and Chytridiomycota. The relative abundance of Ascomycota in the samples acclimated to 40°C reached 92 and 82% after the heat shock in the NW and SE soils, respectively.

## Discussion

### Impact of Warming on Microbial Biomass and Functions

Our short-term (39 days) warming experiment revealed a divergent influence of increased temperatures on microbial diversity, community structure and functioning that was mainly attributable to the soil origin (soil layer and aspect). Gradually increasing temperatures with a subsequent heat shock induced strong changes in microbial community structures and functioning in active layer soils from both south-east and north-west aspects. In contrast, in opposition to our expectations (first hypothesis) permafrost soils showed only minor changes in the microbial community structures and no changes in their functioning, except regarding specific respiration activity (SRA) after the heat shock treatment (Figure 2 and Supplementary Tables S1, S2).

There are several potential explanations for the weak response of the permafrost microbiome to warming and heat shock. One possibility is the short incubation period (e.g., only partial reactivation of dormant permafrost spores, e.g., *Fonticella* OTU within Firmicutes, Figure 5), but a deficiency of energy and nutrients available for enzymatic degradation is more probable. Čapek et al. (2015) found that the degradation of autochthonous soil carbon by the permafrost microbiome was not sufficient to induce an increase in the microbial biomass of Siberian permafrost. The authors showed that the availability of allochthonous sources from the top soil might have accelerated microbial growth. This most likely explains why permafrost soils responded to warming only weakly compared to the active layer soils in our study, even though all soils had comparable DOC and DN contents (Table 1 and Supplementary Tables S1, S2). Low-quality nutrients require a high amount of energy to be degraded (Čapek et al., 2015) and therefore may not be accessible for microorganisms in deep soil layers such as permafrost (160 cm depth in our study), which are considered energy limited (Fontaine et al., 2007; Wild et al., 2014).

DNA content, considered as a proxy for microbial biomass, did not show substantial changes under warming until 25°C, regardless of the soil origin (Supplementary Table S1). However, we observed a strong decrease in DNA content at 40°C in the active layer soils, in line with findings from other heat stress studies with alpine meadow, arable, and forest soils (Chaer et al., 2009; Riah-Anglet et al., 2015). A soil temperature of 40°C seems to be excessively high and has been used to induce drastic changes in soil microbial communities in order to improve the knowledge on community structure–function relationships (Riah-Anglet et al., 2015). Nevertheless, we chose 40°C based on previous measurements of soil temperature on south-exposed mountainous soils (Zumsteg et al., 2013). Surprisingly, 40°C did not influence the microbial biomass (DNA content) of PF soils (Supplementary Table S1), and the decrease observed in the active layers was more pronounced in NW soils than in SE soils, suggesting a higher tolerance of the microbial communities to higher temperatures in south-east exposed soils which is in agreement with our second hypothesis. This result also supports the stronger increase in SRA in soils from the north-west exposed slope, which typically experiences a narrower range of temperatures during the year and a lower annual mean temperature.

Consistent with our results, it has been argued that SRA strongly responds to short-term warming of soils (Davidson and Janssens, 2006; Kirschbaum, 2006). An increase in SRA in the NW and SE active layer soils with increased temperatures up to 25°C might be attributable to a shift to a higher demand for microbial C maintenance which otherwise would lead to a drop in microbial biomass (Anderson and Domsch, 2010). Therefore, a reduction in DNA content (microbial biomass), which we observed simultaneous to an increase in SRA, at 40°C indicates a lack of energy to sustain growth of soil microorganisms at this high temperature. We also found a shift from more complex to easily available C sources in our Biolog assay (Supplementary Figure S1), reflecting the effects of elevated temperature on metabolic potential, which is consistent with results from an earlier study by Wu et al. (2015). Microorganisms may be able to physiologically adjust and decrease their SRA under long-term warming via various biochemical adaptations (Allison and Martiny, 2008; Bradford et al., 2010), and acclimation may take days to weeks (Hochachka and Somero, 2002). In our study, acclimation to increased temperatures up to 25°C was followed by a 40°C heat shock, and subsequently by a temperature decrease and stabilization at the original temperature of 4°C. After the heat shock, SRA in both active layer soils declined back to the initial values but the biomass remained very low and the microbial community shifted significantly to a new structure with reduced diversity (Figure 3 and Supplementary Tables S1, S2). It is debatable whether this response should be interpreted as a type of microbial adaptation to the new stress condition (Allison and Martiny, 2008) or as just a simple consequence of substrate deficiency (Kirschbaum, 2004; Hartley et al., 2008). We suggest that the microorganisms in the active layer soils shifted to a simpler and more stress resistant and resilient microbial community (Allison and Martiny, 2008; Wu et al., 2015) thriving under conditions of reduced substrate availability. We further suggest that the increase in SRA between 4 and 25°C, which was not accompanied by a drop in microbial biomass, indicates substrate limitation, while the increase in SRA in active layer soils at 40°C, connected with a drop in microbial biomass, rather implies temperature stress. We therefore suggest that the maximum recorded soil temperature of 29.6°C at our study sites (on the south-east exposed slope at 3000 m a.s.l.) is close to the temperature threshold below which the soil microbial communities in active layers can still withstand temperature stress with no or only minor changes in microbial functioning.

### Impact of Warming on Microbial Diversity and Community Structure

A gradual warming (4–25°C) of NW and SE active layer soil samples, slightly increased bacterial alpha-diversity, which is in line with other studies in cold regions (Yergeau and Kowalchuk, 2008; Xiong et al., 2014b; Wu et al., 2015). In contrast, the 40°C treatment strongly reduced bacterial alpha-diversity in all soils, with NW soils experiencing the strongest reduction in alpha-diversity. NW soils had the highest alpha-diversity and were adapted to a lower temperature (annual mean soil temperature at 5 cm soil depth of −1.6°C) than SE soil (annual mean soil temperature at 5 cm soil depth of +1.7°C). Extreme temperatures up to 50°C will kill many of the original microorganisms, enabling colonization by other microorganisms adapted to growth at higher temperatures (Bárcenas-Moreno et al., 2009). Interestingly, the drop in alpha-diversity in the heat shock treatment (40°C) was more pronounced in bacteria than fungi, suggesting higher sensitivity of bacteria to temperature stress (large temperature changes) than fungi with respect to the number of OTUs, which is in accordance with findings from Xiong et al. (2014a) that warming did not significantly alter fungal alpha-diversity.

Surprisingly, microbial communities in permafrost soils were less responsive to elevated temperatures than those in active layer soils. We expected to find the opposite pattern because microbial communities in the active layer soils, in particular those from the south-east exposed slope, experience larger temperature fluctuations under field conditions than permafrost soils. The delayed or lack of response of the permafrost microbiome to warming might be due to the low metabolic rate of cold-adapted bacteria, as documented for Antarctic soils (Yergeau et al., 2012; de Scally et al., 2016; Chong et al., 2018) or to deficiency of energy and nutrients (see above). Another explanation could be that the incubation time of 39 days was too short to break of the dormant state of bacterial spores commonly found in permafrost soils (Frey et al., 2016).

Similar to alpha-diversity, all changes in bacterial and fungal community structures were more pronounced for bacteria than for fungi, independent of the soil origin. Shifts in microbial communities have often been detected in other warming studies (Zhou et al., 2012; Xiong et al., 2014b; Wu et al., 2015). Consistent with our findings, fungi have been observed to be less sensitive to temperature than bacteria (Yuste et al., 2011; Xiong et al., 2014a). Greater resistance of fungi to warming has been explained by their need for several years to respond to temperature changes (Rinnan et al., 2007; Xiong et al., 2014a), which could be a result of their higher tolerance to environmental changes compared to bacteria (De Vries and Shade, 2013; Sun et al., 2017). This higher tolerance of fungi may be because their networks are more stable than bacterial networks (de Vries et al., 2018). In addition, it has been suggested that fungi are more resistant than bacteria to moisture and temperature changes, owing to their chitinous cell walls (Holland and Coleman, 1987). In contrast, metatranscriptomic profiling revealed that fungal activities quickly increased to permafrost thaw (Coolen and Orsi, 2015). One reason for the discrepancy of responses between our study and their studies could be the different age, origin and nature of the permafrost. In our experiment, we have used holocene permafrost with low TOC and TN content whereas in their study the permafrost contained a significant input of plant-derived carbon as a source of energy and nutrients to break down SOM (Čapek et al., 2015), which might explain the immediate response of fungi after permafrost thaw. Heat shock led to the most pronounced shift in community structure, independent of acclimation temperature, as shown in the CAP ordination where bacterial and fungal community structures in all soils at 40°C clustered away from the other acclimation temperatures (Figure 4).

Overall, warming had strong effects on the abundances of several taxa that are considered indicators of temperature change. The most responsive taxa of both the NW and SE active layer soils differed considerably from those of the PF soils. In the PF soils there was an increase in the relative abundance of Proteobacteria and Bacteroidetes taxa with increasing temperature, in accordance with the findings of Xiong et al. (2014b). These phyla are considered to contain a number of copiotrophic taxa, which are known to be positively correlated with soil-available C pools and able to growth rapidly in the presence of labile soil carbon substrates (Fierer et al., 2007). Such a change suggests an ongoing slow release of available nutrients in the otherwise energy-poor permafrost soils, which may be induced by the slow release of previously frozen substrate or by the decomposition of dead biomass after the heat shock. In combination with the decrease in Acidobacteria (reaction of the different subphyla), also observed for our PF soils, this shift may suggest an expansion of copiotrophs at the expense of oligotrophs (Fierer et al., 2007).

Bacteroidetes is a bacterial phylum, commonly considered to feature a copiotrophic lifestyle (Fierer et al., 2007), that showed an interesting pattern in our study. OTUs represented mainly by *Mucilaginibacter* in the PF soils increased with increasing temperatures, whereas OTUs affiliated with *Mucilaginibacter* and Sphingobacteriales in the active layer soils showed the opposite pattern (Figure 5). This indicates that OTUs within the same taxonomic group can react differently to increasing temperatures in permafrost and active soil layers. Similarly, Xiong et al. (2014b) reported that the relative abundance of Bacteroidetes was not significantly altered by elevated temperature, whereas nearly all OTUs within Sphingobacteria had a significantly lower abundance. Members of Sphingobacteria are common in soils (Janssen, 2006) and have also been found in diverse environments including deserts. They can use a wide variety of organic compounds as sources of carbon and energy (Aslam et al., 2016).

A more striking finding of our temperature experiments was that Firmicutes, mainly represented by *Tumebacillus* and other OTUs within Alicyclobacillaceae, strongly increased at 40°C in NW and SE soils, even reaching 50% of the total abundance in the NW soils. The genus *Tumebacillus*, a member of the family Alicyclobacillaceae, has been isolated from diverse environments including Arctic permafrost, soil and wastewater (Kim and Kim, 2016). Members of Firmicutes have showed dominance in previous heat-shock experiments (Kannaiah Goud et al., 2014; Mei et al., 2016). Several taxa within Firmicutes are endospore formers, and this property makes them hardy in potentially harsh conditions (Filippidou et al., 2015).

Across all soil samples a significant decrease in Deltaproteobacteria and Saccharibacteria with elevated temperatures was observed. Deltaproteobacteria, mainly represented by Bdellovibrio, did not respond to increasing temperatures until 25°C but then strongly decreased in relative abundance at 40°C, in line with findings of Wu et al. (2015). Similarly, a significant decrease in Saccharibacteria with elevated temperatures was observed in our study. Saccharibacteria (TM7) have been observed previously in permafrost soils from the same site (Frey et al., 2016). In a recent study using single-cell genomics, genomes were obtained from uncultured bacteria within this phylum (Starr et al., 2018). Genomes of Saccharibacteria contain genes for complex carbon utilization and amino acid metabolism making them important members of the permafrost microbiome.

In our study, Ascomycota increased in relative abundance with elevated temperatures in PF and SE soils at 40°C, whereas Basidiomycota showed only a significant increase at the phylum level in the NW soils at 40°C (Supplementary Table S4). Xiong et al. (2014a) reported an increase in Basidiomycota after warming. In our study, Basidiomycetous yeasts (*Cryptococcus*), together with Mucoromycota (*Mortierella*), were favored by warming in permafrost soils (Figure 6). In general, the fungal community in the permafrost layer was characterized by these taxa with a copiotrophic lifestyle (Frey et al., 2016). These fungal groups are common in cryosphere environments because they have developed successful structural and functional adaptations (e.g., spore formation, membrane modification, production of cryoprotectants, and synthesis of enzymes operating at low temperatures) not only to survive but also to be metabolically active and propagate under extremely cold and nutrient-poor conditions (Ozerskaya et al., 2009; Buzzini et al., 2012).

Dothideomycetes (mainly Pleosporales) tended to increase with increasing temperature, with lower soil temperature at the soil origin corresponding to a greater increase in relative abundance (Figure 6). These fungi often occur in oligotrophic environments and are known to be tolerant to desiccation and osmotic stress (Knapp et al., 2012). Interestingly, within Eurotiomycetes different taxa responded divergently to increasing temperatures. In both active layer soils (SE and NW), *Aspergillus* increased strongly in relative abundance. *Aspergillus* is a pathogenic fungus able to withstand extreme environmental conditions. In contrast, Verrucariaceae decreased in all soils under increased temperatures, indicating that fungi within a taxonomic group can give heterogenous responses to warming. Overall, taxonomically heterogeneous responses are not surprising, as fungal species are known to vary strongly in their carbon resource niches (Baldrian, 2009) and have developed specific adaptation mechanisms to cope with elevated temperature, desiccation, starvation and other abiotic stresses (Sun et al., 2017).

Overall, the relationship between microbial and functional shifts and warming is still not clear (Riah-Anglet et al., 2015) and may depend on local conditions. Contrary to our expectations (our third hypothesis), we did not observe significant differences between bacteria and fungi in the explained variability, and we observed a substantial influence of the soil origin on the link between shifts in microbial community structures and functional changes in the soil. We found that changes in microbial community structures with warming are linked more strongly to changes either in carbon use patterns or in enzyme activities, depending on the original soil temperature. The higher the soil temperature at the site of soil origin (SE > NW > PF), the greater the changes are, which is in accordance with the findings of Wu et al. (2015). On the other hand, this relationship has been reported to be diversity-dependent in other studies (Griffiths et al., 2000; Riah-Anglet et al., 2015). In our investigation, PF soils with permanent low temperature under field conditions (−0.8°C) did not show a significant link (Supplementary Figure S2) between these variables, indicating that changes in microbial community structures are not necessarily related to microbial functions. In contrast, the active layer soils exhibited a link between the community shifts and functional changes (carbon use patterns and enzyme activities) with warming. Therefore, these soils could already be actively responding to the warming based on their previous experience at the field site; this is especially likely for the soils from the south-east exposed slope, which displayed the strongest relationships. This trend may indicate large differences in the microbial adaptability to temperature changes, even within a small distance, in high-alpine environments, supporting the importance of including this heterogeneity of soil origin in climate models. It seems that, at least in the short term, microbial communities shaped by a different temperature range present distinct abilities to deal with temperature stress. However, long-term responses must additionally be investigated to determine whether these changes are only temporary.

## Conclusion

In conclusion, we observed that microbial community structures exhibited distinct responses of fungal and bacterial communities to temperature perturbations of different magnitudes. They showed relatively high functional stability until 25°C, a temperature that we suggest is close to a threshold of the community stress tolerance, but strong changes in community structures and microbial functioning at 40°C. Permafrost communities were surprisingly resistant to marked increases in temperature. Different microbial taxa showed inconsistent responses to elevated temperature due to conditional dependencies between taxonomic groups (e.g., competition between a given pair of taxa may rapidly increase/decrease above a particular environmental threshold). In agreement with findings from other studies, fungi were highly resistant to increased temperatures compared to bacteria. It is possible that fungal communities require more time (up to years) to respond to warming manipulation or fungi possess specific adaptation mechanisms such as desiccation and starvation to cope with elevated temperature. The overall relationship between the community shifts and functional changes closely reflected the original temperatures of the soils in the field. The slope aspect played a defining role in the response of the microbial community, as the warm-adapted south-west facing slope was more tolerant to a higher temperature regime. A sudden heat shock (40°C) event had significant effects on many phyla, which caused decrease SRA and alpha-diversity. Reactions of microbial community structures to a heat shock depended on the acclimation temperature: uninhibited at 15°C, inhibited with recovery at 25°C, and inhibited without recovery at severe heat shock (acclimation at 4°C). Overall, these observations indicate distinct physiological traits and ecological niches associated with individual microbial groups.

## Data Availability

The datasets generated for this study can be found in NCBI Sequence Read Archive, PRJNA497433.

## Author Contributions

PL and BF designed the study. BF participated in sample collection. PL, CP-M, and JR contributed to data collection. PL, BF, and JR performed the data analysis. PL and BF wrote the manuscript. CP-M and HŠ contributed to the writing.

## Conflict of Interest Statement

The authors declare that the research was conducted in the absence of any commercial or financial relationships that could be construed as a potential conflict of interest.

## Supplementary Material

FIGURE S1 Non-metric multidimensional scaling (NMDS) analyses of Bray-Curtis dissimilarities distances of carbon utilization patterns (Biolog) in **(A)** SE and **(B)** NW soils (active layer). Each ordination shows the combination of constrained carbon sources for each temperature after acclimation and changes after heat shock. Displayed carbon sources shifted significantly from recalcitrant to more easily decomposable ones after exposure to 40°C: NW (pseudo-F_PERMANOVA_ = 3.65, *p* < 0.001) and SE (pseudo-F_PERMANOVA_ = 7.71, *p* < 0.001).

FIGURE S2 Relationship between microbial community structure and functional parameters (C utilization patterns and potential enzyme activity) displayed by the *R*^2^ values of linear regressions run separately for bacteria and fungi. Vertical lines represent bootstrapped 95% confidence intervals and asterisks represent the significance of the regressions (^∗^significant; NS, not significant).

TABLE S1 Effects of soil origin, temperature and their interactions on DNA content, specific respiration activity (SRA) and potential enzymatic activities.

TABLE S2 Effects of soil origin, temperature, and their interactions on bacterial and fungal alpha-diversity.

TABLE S3 Effects of soil origin, temperature, and their interactions on the most abundant bacterial taxa (phyla or class).

TABLE S4 Effects of soil origin, temperature, and their interactions on the most abundant fungal taxa.
