# Supplementary figures and images for: High-Alpine Permafrost and Active-Layer Soil Microbiomes Differ in Their Response to Elevated Temperatures
Source: Front Microbiol. 2019 Apr 3;10:668. doi: 10.3389/fmicb.2019.00668 (PMC6456652; doi:10.3389/fmicb.2019.00668)

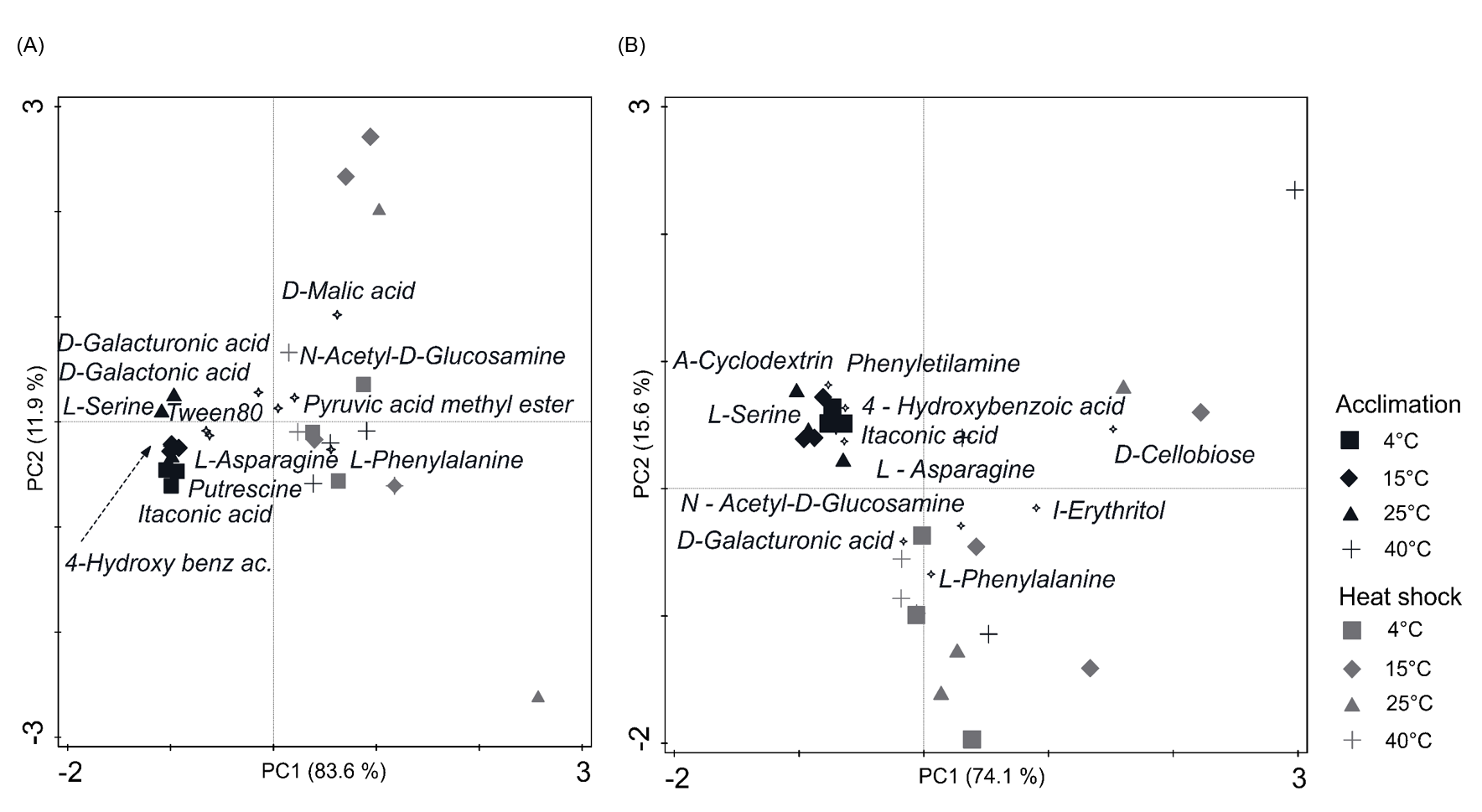

Supplement: FIGURE S1 — Non-metric multidimensional scaling (NMDS) analyses of Bray-Curtis dissimilarities distances of carbon utilization patterns (Biolog) in (A) SE and (B) NW soils (active layer). Each ordination shows the combination of constrained carbon sources for each temperature after acclimation and changes after heat shock. Displayed carbon sources shifted significantly from recalcitrant to more easily decomposable ones after exposure to 40°C: NW (pseudo-FPERMANOVA = 3.65, p < 0.001) and SE (pseudo-FPERMANOVA = 7.71, p < 0.001). [file Image_1.TIF]

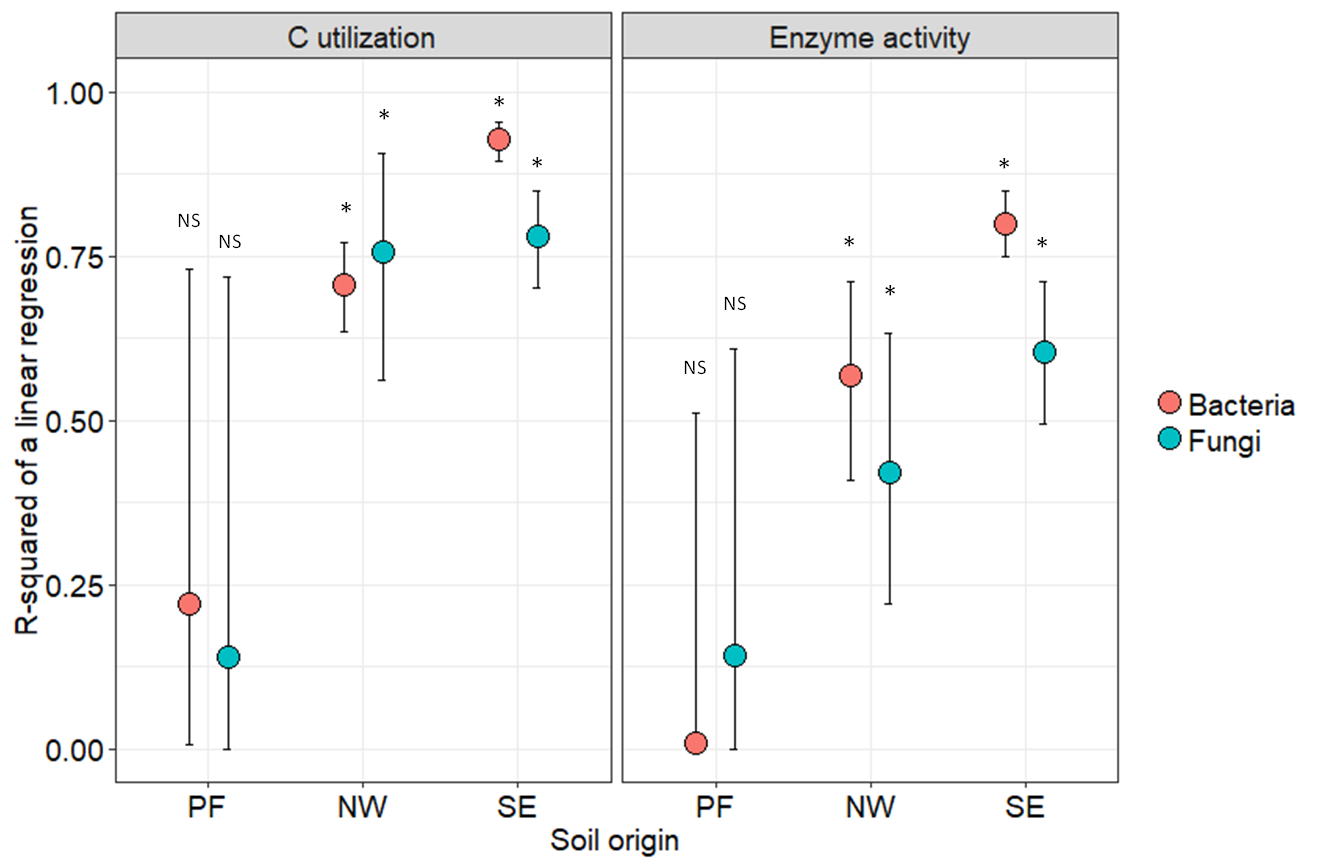

Supplement: FIGURE S2 — Relationship between microbial community structure and functional parameters (C utilization patterns and potential enzyme activity) displayed by the R2 values of linear regressions run separately for bacteria and fungi. Vertical lines represent bootstrapped 95% confidence intervals and asterisks represent the significance of the regressions (∗significant; NS, not significant). [file Image_2.TIF]
